# Common origin of the coronary arteries from the right sinus with intramyocardial course of the anterior descent artery

**DOI:** 10.31744/einstein_journal/2020AI5174

**Published:** 2019-11-08

**Authors:** Eduardo Kaiser Ururahy Nunes Fonseca, Lucas de Pádua Gomes de Farias, Bruna Melo Coelho Loureiro, Daniel Giunchetti Strabelli, Nevelton Heringer, Luiz Francisco Rodrigues de Ávila

**Affiliations:** 1 Heart Institute, Hospital das Clínicas, Faculdade de Medicina, Universidade de São Paulo, São Paulo, SP, Brazil.

A 62-year-old patient referred to coronary angiotomography due to ischemic changes detected in an exercise stress test. The patient reported hypertension and dyslipidemia, both controlled with drug treatment and regular physical exercise. She didn’t have any cardiovascular complaints and her physical examination was unremarkable.

**Figure 1:**
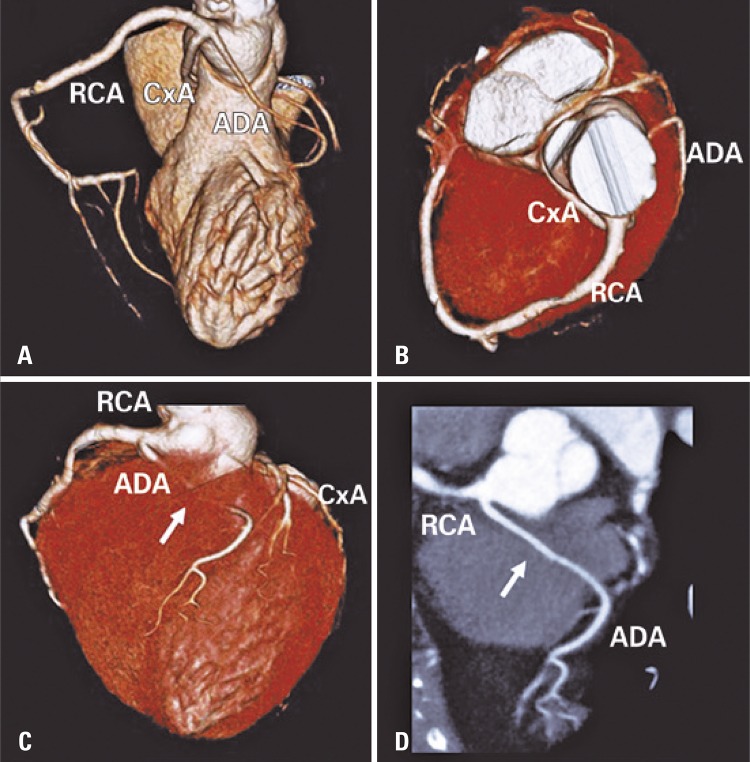
Tridimensional volumetric reconstruction. Right lateral view (A), cranial view (B), anterior view (C) and multiplanar reconstruction (D) of coronary angiotomography showing the common origin of coronary arteries through a short common trunk, emerging from right Valsalva sinus and trifurcating into right coronary artery, circumflex artery, anterior descending artery, and highlighting both an intra-arterial course of anterior descending artery, between aorta and pulmonary trunk, and a long proximal intramyocardial course (arrow) RCA: right coronary artery; CxA: circumflex artery; ADA: anterior descending artery.

Coronary angiotomography showed common origin of coronary arteries through a short common trunk, emerging from the right Valsalva sinus and trifurcating into right coronary artery (RCA), circumflex artery (CxA) and anterior descending artery (ADA). The RCA had habitual course and gives rise to the posterior descending artery (right-dominance). The CxA had a small caliber and retroaortic course without luminal. The ADA had an important angulation in its origin, interarterial course in subvalvar plan and a long proximal intramyocardial segment, where it exhibited a reduction of its diameter, without plaques or parietal changes ( Figure 1 ).

Anomalies of origin and coronary course are relatively common findings,^1^ and they can generate ischemia or have no clinical significance. The coronary angiotomography is the goldstandard for anatomy assessment showing coronary courses and their myocardial relationship,^1 - 6^ elegantly depicted by volumetric reconstructions. Among described anomalies, reports about common origin of the coronary arteries by single trunk from the right Valsalva sinus are rare.^2 - 4^ To the best of our knowledge, the association of this set of findings with a long intramyocardial course of the anterior descending artery, determining local vessel compression in ideal conditions of exam (diastolic acquisition with vasodilation and heart rate control) was not previous reported in the published literature, therefore, this is the first case to be reported.
