# Supplementary material for: Common origin of the coronary arteries from the right sinus with intramyocardial course of the anterior descent artery
Source: Einstein (Sao Paulo). 2019 Nov 8;18:eAI5174. doi: 10.31744/einstein_journal/2020AI5174 (PMC6896596; doi:10.31744/einstein_journal/2020AI5174)
Supplement: Translation

# Translation: Origem anômala comum coronariana do seio direito com trajeto intramiocárdico da artéria descendente anterior

**Authors:** Eduardo Kaiser Ururahy Nunes Fonseca, Lucas de Pádua Gomes de Farias, Bruna Melo Coelho Loureiro, Daniel Giunchetti Strabelli, Nevelton Heringer, Luiz Francisco Rodrigues de Ávila

**Affiliations:** 1 Instituto do Coração, Hospital das Clínicas, Faculdade de Medicina, Universidade de São Paulo, São Paulo, SP, Brasil.

Paciente, 62 anos, encaminhada para estudo angiotomográfico das coronárias devido a alteração isquêmica detectada em teste de esforço de rotina. Relatava hipertensão e dislipidemia, ambas controladas sob tratamento medicamentoso, e prática regular de atividade física. Sem queixas cardiovasculares ou alterações ao exame físico.

Angiotomografia coronária evidenciou origem comum das artérias coronárias através de tronco comum de curto trajeto, emergindo do seio de Valsalva direito e trifurcando-se em coronária direita (CD), circunflexa (CX) e descendente anterior (DA). A CD com trajeto habitual, dominante, apresentava ramo descendente posterior precoce. A CX apresentava pequeno calibre, trajeto retroaórtico, sem redução luminal. Descendente anterior apresentava importante angulação em sua origem, trajeto interarterial no plano subvalvar, apresentando extenso segmento proximal intramiocárdico, onde exibia afilamento fisiológico de seu calibre, sem placas ou alterações parietais ( Figura 1 ).

**Figura 1:** Reconstrução tridimensional volumétrica. Vista lateral direita (A), superior (B) e anterior (C), e reconstrução multiplanar (D) de angiotomografia de coronárias evidenciam origem comum das artérias coronárias no seio coronariano direito através de tronco comum de curto trajeto, emergindo do seio de Valsalva direito e trifurcando-se em artéria coronária direita, artéria circunflexa e artéria descendente anterior, com destaque para o trajeto interarterial da artéria descendente anterior, entre a aorta e o tronco principal da artéria pulmonar, bem como longo trajeto intramiocárdico do seu segmento proximal (seta) CD: artéria coronária direita; CX: artéria circunflexa; DA: artéria descendente anterior.

As anomalias de origem e trajeto coronarianos são achados relativamente frequentes,^1^ podendo gerar isquemia ou serem desprovidos de significado clínico. A angiotomografia das coronárias é o padrão-ouro na avaliação dessa anatomia,^1 - 6^ demonstrando com elegância, por meio de reconstruções volumétricas, todo o trajeto vascular e sua relação com o miocárdio. Dentre as anomalias descritas, raros são os casos relatados de origem comum das coronárias por meio de tronco único saindo do seio de Valsalva direito.^2 - 4^ Até onde sabemos, a associação desse conjunto de achados com trajeto intramiocárdico extenso da artéria descendente anterior, determinando afilamento vascular local já em condições ideais de exame (aquisição diastólica com vasodilatador e betabloqueador) não possui precedente na literatura, sendo a primeira descrição do tipo.
